# Correlation of Milestone Restricted Mean Survival Time Ratio With Overall Survival Hazard Ratio in Randomized Clinical Trials of Immune Checkpoint Inhibitors: A Systematic Review and Meta-analysis

**DOI:** 10.1001/jamanetworkopen.2019.3433

**Published:** 2019-05-03

**Authors:** Zi-Xian Wang, Hao-Xiang Wu, Li Xie, Ying-Nan Wang, Lu-Ping Yang, Ming-Ming He, Hui-Yan Luo, Pei-Rong Ding, Dan Xie, Gong Chen, Yu-Hong Li, Feng Wang, Rui-Hua Xu

**Affiliations:** 1 Department of Medical Oncology, State Key Laboratory of Oncology in South China, Collaborative Innovation Center for Cancer Medicine, Sun Yat-Sen University Cancer Center, Guangzhou, China; 2 Faculty of Medical Sciences, Sun Yat-Sen University, Guangzhou, China; 3 Clinical Research Center, Shanghai Jiao Tong University School of Medicine, Shanghai, China; 4 Department of Colorectal Surgery, State Key Laboratory of Oncology in South China, Collaborative Innovation Center for Cancer Medicine, Sun Yat-Sen University Cancer Center, Guangzhou, China; 5 Department of Pathology, State Key Laboratory of Oncology in South China, Collaborative Innovation Center for Cancer Medicine, Sun Yat-Sen University Cancer Center, Guangzhou, China

## Abstract

**Importance:**

Immune checkpoint inhibitors (ICIs) have unique patterns of response and survival that differ from conventional chemotherapies. Novel intermediate end points are urgently required to detect the early signals of ICI activity.

**Objective:**

To evaluate milestone rate (Kaplan-Meier estimates of survival probabilities at given time points) and milestone restricted mean survival time (RMST, the area under survival curves up to given time points) as potential intermediate end points for ICI trials.

**Data Sources:**

Electronic databases (pre-MEDLINE, MEDLINE, Embase, and the Cochrane Central Register of Controlled Trials) were searched for randomized clinical trials published between January 1, 2000, and December 31, 2017.

**Study Selection:**

Phase 2 and phase 3 randomized clinical trials evaluating ICIs in advanced solid tumors.

**Data Extraction and Synthesis:**

Two investigators extracted the data and reconstructed individual patient data to estimate the milestone rate or milestone RMST from the published Kaplan-Meier curves.

**Main Outcomes and Measures:**

Trial-level milestone rates or milestone RMSTs were estimated for 6-month and 9-month progression-free survival (PFS) and 9-month and 12-month overall survival (OS). A weighted linear regression model evaluated the correlations of ratios of milestone rates or milestone RMSTs with OS hazard ratios (HRs).

**Results:**

Twenty-six trials examining 8 different tumor types were identified, including 12 892 patients. Overall survival HR was correlated with the ratio of 9-month OS milestone rate (*R*^2^ = 0.45; 95% CI, 0.27-0.74), and with the ratio of 12-month OS milestone rate (*R*^2^ = 0.40; 95% CI, 0.22-0.70). The ratio of 9-month OS milestone RMST (*R*^2^ = 0.60; 95% CI, 0.28-0.74) and ratio of 12-month OS milestone RMST were correlated with OS HR (*R*^2^ = 0.64; 95% CI, 0.42-0.78). No correlations were observed between OS HR and the ratio of 6-month or 9-month PFS milestone rates or milestone RMSTs.

**Conclusions and Relevance:**

Ratios of OS milestone RMSTs had a stronger correlation with OS HRs than ratios of OS milestone rates, whereas ratios of PFS milestone rates and ratios of PFS milestone RMSTs were not correlated with OS HRs. The OS milestone RMST could be further studied as an intermediate end point in future ICI trials.

## Key Points

**Question:**

What intermediate end points could be useful in randomized clinical trials studying immune checkpoint inhibitors?

**Findings:**

In this systematic review and meta-analysis of 26 trials studying immune checkpoint inhibitors in 12 892 participants, the ratio of milestone restricted mean survival time for overall survival was more strongly correlated with the overall survival hazard ratio than the ratio of overall survival milestone rates.

**Meaning:**

Milestone restricted mean survival time could be studied as a potential intermediate end point for overall survival in future trials of immune checkpoint inhibitors.

## Teaser

This systematic review and meta-analysis examines randomized clinical trials (RCTs) of immune checkpoint inhibitors to evaluate the use of milestone rate and milestone restricted mean survival time as intermediate end points in immune checkpoint inhibitor trials.

## Introduction

There have been major advances in cancer immunotherapies in the last 10 years, among which the most promising approach is the use of immune checkpoint inhibitors (ICIs) to activate self-immunity toward tumors.^1^ The broad efficacy of ICIs for various malignant tumors has led to unprecedented levels of research and development of these agents.^2^ Meanwhile, a unique pattern of response, progression, and survival among patients who received ICIs that differs from conventional chemotherapy or targeted therapy has stimulated concern.^3^ The delayed clinical effects and long-term survival demonstrated by ICI treatments could lead to substantially extended study duration and loss of statistical power if these 2 unique features are not accounted for in the study design and statistical analyses.^4^ Additionally, several previous studies have shown no association of the odds ratio for the objective response rate or the progression-free survival (PFS) hazard ratio (HR) with the overall survival (OS) HR, raising questions for the use of these traditional surrogate end points in ICI trials.^5,6,7^

With the statistical issues and challenges posed by ICIs, researchers are investigating potential intermediate end points for ICI trials, including milestone survival, defined as the Kaplan-Meier estimate of survival probability at a given time point.^8^ A 2017 study of milestone survival^9^ suggested that the 12-month OS rate could be used as a potential intermediate end point in ICI trials for non–small cell lung cancer (NSCLC). However, that investigation was based on the analysis of trials pooled by different therapeutic classes, including conventional therapy, targeted therapy, and immunotherapy. Furthermore, drawbacks of milestone survival include the cross-sectional assessment at a given time point, the inability to account for the totality of the survival period, and the effect of censoring before the milestone time point.

The restricted mean survival time (RMST) is an alternative treatment outcome measure that can be estimated as the area under the survival curve up to a prespecified time horizon and hence can account for all survival information before that time horizon.^10,11^ In this study, we used RMST to measure milestone treatment effect and assessed ratios of milestone RMSTs against ratios of milestone rates as a potential intermediate end point for ICI trials.

## Methods

This study was conducted in compliance with the recommendations of the Cochrane Handbook for Systematic Reviews of Interventions and reported based on Preferred Reporting Items for Systematic Reviews and Meta-analyses (PRISMA) reporting guideline.

### Selection of Randomized Trials

We searched pre-MEDLINE, MEDLINE, Embase, and the Cochrane Central Register of Controlled Trials for randomized clinical trial results published from January 1, 2000, to December 31, 2017. Analysis began in June 2018. We combined Medical Subject Headings and free-text terms to identify relevant studies. The full search strategy is detailed in the eMethods in the Supplement. Inclusion criteria included reports of randomized clinical trials of ICIs that included a Kaplan-Meier curve for OS. Phase 1, single-arm phase 2, dose-finding, and adjuvant setting trials were excluded. News, editorials, letters, commentaries, retrospective studies, review articles, and secondary analyses of randomized clinical trials were also excluded. For multiple-arm trials, all comparisons were included. The intention-to-treat population was included except for 1 trial, in which the population with programmed cell death 1 (PDCD1) ligand 1 expression in 5% or more of tumor-infiltrating immune cells was selected according to the trial design.^12^

### Data Extraction and Reconstruction of Individual Patient Data

Two of us (Z-X.W. and H-X.W.) screened the trials independently for eligibility and extracted the following information from each included trial: first author’s name, year of publication, tumor type, treatment regimen in both arms, phase and treatment line of the trial, primary or coprimary end point, minimum follow-up, and sample size. Any discrepancies were resolved by consensus.

To estimate the milestone survival rate and milestone RMST, we reconstructed individual patient data for each group from the published Kaplan-Meier curves.^13^ DigitizeIt software version 2.2 (DigitizeIt) was used to measure the time and survival probability coordinates on the Kaplan-Meier curves. The number of patients at risk and the total number of events were extracted when available. Next, the data were entered into an algorithm on the basis of iterative numerical methods to solve the inverted Kaplan-Meier equations. All data were reconstructed by one of us (H.-X.W.) and validated by another (Z.-X.W.).

### Outcome Measurement

Four trial-level milestones were chosen for analysis: 6-month PFS, 9-month PFS, 9-month OS, and 12-month OS. We calculated the milestone rates using Kaplan-Meier estimates. The milestone ratio was defined as the ratio of milestone rates between 2 treatment arms. We assessed the milestone RMSTs in the experimental and control groups by prespecifying the time horizon as 6, 9, or 12 months.^14,15^ The milestone RMST from the Kaplan-Meier estimate of the survival function was determined, and the ratio of milestone RMST was estimated. The associated variances were estimated using the δ method.^16^

### Statistical Analysis

Nonproportional hazards were tested using the Grambsch-Therneau test, and a 2-tailed *P* less than .10 indicated a statistically significant violation of the proportional hazard assumption.^17^ By pooling the reconstructed individual patient data of the included ICI trials, Kaplan-Meier analyses of the treatment arm vs the control were performed to investigate the survival kinetics among the pooled cohort.

The correlations of treatment effects measured by the ratios of the milestone rate or milestone RMST with the HR were evaluated using weighted linear regression models, with weights equal to the sample size of each randomized comparison. The coefficient of determination (*R*^2^) and 95% CIs from the weighted linear regression model were used to measure strength of the correlations. The 95% CIs of *R*^2^ were obtained using the bootstrap method with 1000 replications. *R*^2^ equal to 0.80 was chosen prospectively as the cutoff value to establish the milestone rate or RMST as validated intermediate end points for the OS HR.^5^

To assess whether any trial was more influential in the trial-level correlation of the OS HR with the ratio of 9-month or 12-month OS milestone RMST, a leave-1-out cross-validation was performed by excluding 1 comparison at a time. Statistical analyses were performed using R statistical software version 3.5.1 (R Project for Statistical Computing), and the survRM2 package was used to derive the milestone RMST.

## Results

**Figure 1.**
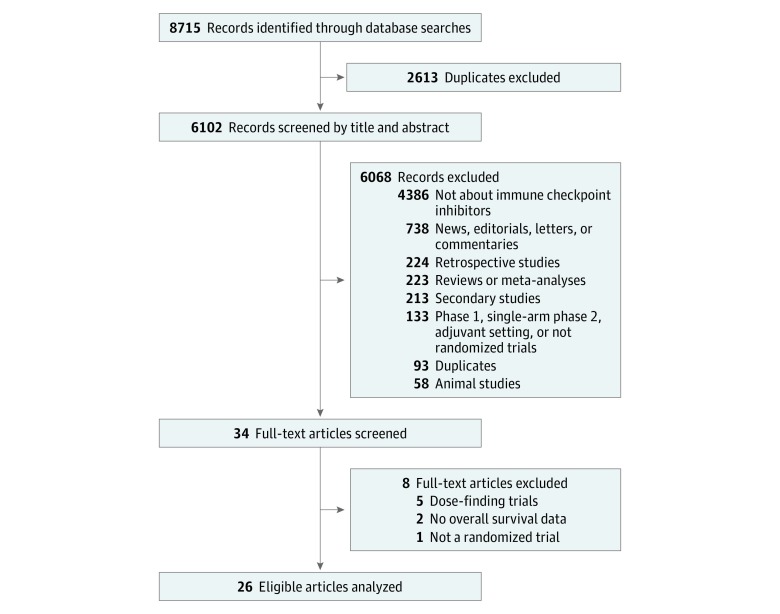
PRISMA Flowchart of Study Inclusions and Exclusions

Twenty-six eligible randomized clinical trials studying ICIs were identified (Figure 1 and Table), including 31 treatment comparisons and 12 892 patients. Twenty trials (77%) were phase 3 studies, and 6 trials (23%) were phase 2 studies. The 26 trials examined 8 tumor types, including 9 on NSCLC (35%) and 8 on melanoma (30%). There were 12 trials (46%) that examined PDCD1 inhibitor monotherapy (8 with nivolumab and 4 with pembrolizumab), 3 trials (12%) of PDCD1 ligand 1 inhibitor monotherapy (atezolizumab), 3 trials (12%) of cytotoxic T lymphocyte–associated antigen 4 inhibitor monotherapy (2 with ipilimumab and 1 with tremelimumab), 7 trials (27%) of a checkpoint inhibitor and chemotherapy or vaccine combination (4 with ipilimumab and chemotherapy, 2 with ipilimumab and vaccine, and 1 with pembrolizumab and chemotherapy), and 1 trial of a PDCD1 inhibitor and cytotoxic T lymphocyte–associated antigen 4 inhibitor combination (nivolumab and ipilimumab). Twelve trials (46%) were first-line studies, and the remaining 14 trials (54%) were second-line studies or beyond. Most of the studies set OS as the primary or coprimary end point (20 [77%]). In most of the trials, PFS was determined by Response Evaluation Criteria in Solid Tumors (RECIST) version 1.1.^43^ At the time of the database lock of each trial, the minimum follow-up duration range was 4.63 months to 39.60 months with a median (interquartile range) of 12.17 (7.80-18.22) months. Detailed regimen information and other trial characteristics are summarized in eTable 1 in the Supplement. eTable 2 and eTable 3 in the Supplement present the reconstructed individual patient data demonstrating estimates that are close to the HRs or median survival times reported in the original articles.

**Table.** Summary of Trials Included

Trial	Tumor Type	Experimental Arm(s)	Control Arm	Primary End Point	Phase	Line	Minimum Follow-up, mo	No. of Patients
Robert et al,^18^ 2015	Melanoma	Nivolumab	Dacarbazine	OS	3	First	6.17	418
Hodi et al,^19^ 2016	Melanoma	Nivolumab + ipilimumab	Ipilimumab	ORR	2	First	25.1	142
Larkin et al,^20^ 2018	Melanoma	Nivolumab	Physician’s choice chemotherapy	OS/ORR	3	≥Second	26.97	405
Robert et al,^21^ 2015	Melanoma	A: Pembrolizumab once every 2 wk; B: pembrolizumab once every 3 wk	Ipilimumab	OS/PFS	3	First/second	12.17	834
Hodi et al,^22^ 2010	Melanoma	A: Ipilimumab + glycoprotein 100; B: ipilimumab	Glycoprotein 100	OS	3	≥Second	8.43	676
Robert et al,^23^ 2011	Melanoma	Ipilimumab + dacarbazine	Dacarbazine	OS	3	First	37.1	502
Hodi et al,^24^ 2014	Melanoma	Ipilimumab + sargramostim	Ipilimumab	OS	2	≥Second	17.37	245
Ribas et al,^25^ 2013	Melanoma	Tremelimumab	Physician’s choice chemotherapy	OS	3	First	39.6	655
Fehrenbacher et al,^26^ 2016	NSCLC	Atezolizumab	Docetaxel	OS	2	≥Second	13.43	287
Rittmeyer et al,^27^ 2017	NSCLC	Atezolizumab	Docetaxel	OS	3	≥Second	19.57	850
Borghaei et al,^28^ 2015	NSCLC	Nivolumab	Docetaxel	OS	3	Second	15.73	582
Brahmer et al,^29^ 2015	NSCLC	Nivolumab	Docetaxel	OS	3	Second	12.63	272
Carbone et al,^30^ 2017	NSCLC	Nivolumab	Physician’s choice chemotherapy	PFS	3	First	16.3	423
Herbst et al,^31^ 2016	NSCLC	A: Pembrolizumab 2 mg/kg; B: pembrolizumab 10 mg/kg	Docetaxel	OS/PFS	2-3	≥Second	7.17	1034
Langer et al,^32^ 2016	NSCLC	Pembrolizumab + carboplatin +pemetrexed	Carboplatin +pemetrexed	ORR	2	First	6.53	123
Reck et al,^33^ 2016	NSCLC	Pembrolizumab	Physician’s choice chemotherapy	PFS	3	First	6.43	305
Lynch et al,^34^ 2012	NSCLC	A: Concurrent ipilimumab + carboplatin + paclitaxel; B: phased ipilimumab + carboplatin + paclitaxel	Paclitaxel + carboplatin	irPFS	2	First	19.07	204
Kang et al,^35^ 2017	EG	Nivolumab	Placebo	OS	3	Third	5.63	493
Ferris et al,^36^ 2016	HNSCC	Nivolumab	Physician’s choice chemotherapy	OS	3	≥Second	4.63	361
Kwon et al,^37^ 2014	Prostate cancer	Ipilimumab	Placebo	OS	3	Second	11.9	799
Beer et al,^38^ 2017	Prostate cancer	Ipilimumab	Placebo	OS	3	First	24	602
Motzer et al,^39^ 2015	Renal cell carcinoma	Nivolumab	Everolimus	OS	3	Second/third	15.23	821
Reck et al,^40^ 2013	SCLC	A: Concurrent ipilimumab + carboplatin + paclitaxel; B: phased ipilimumab + carboplatin + paclitaxel	Paclitaxel + carboplatin	irPFS	2	First	11.1	130
Reck et al,^41^ 2016	SCLC	Ipilimumab + etoposide + platinum	Etoposide + platinum	OS	3	First	9.4	954
Powles et al,^12^ 2018^a^	Urothelial carcinoma	Atezolizumab	Physician’s choice chemotherapy	OS	3	Second/third	13.07	234
Bellmunt et al,^42^ 2017	Urothelial carcinoma	Pembrolizumab	Physician’s choice chemotherapy	OS/PFS	3	Second	9.97	542

Abbreviations: EG, gastric or gastroesophageal junction cancer; HNSCC, squamous cell carcinoma of the head and neck; irPFS, immune-related progression-free survival; NSCLC, non–small cell lung cancer; ORR, objective response rate; OS, overall survival; PFS, progression-free survival; SCLC, small cell lung cancer

^a^ The population with programmed cell death 1 ligand expression in 5% or less of tumor-infiltrating immune cells was included according to the trial design.

A delayed clinical effect of the ICIs was observed in most trials. When visually assessed, 25 OS curves (81%) and 26 PFS curves (74%) were inseparable in the first 3 months of the 31 examined treatment comparisons. In the pooled cohort of 12 892 patients, the OS curve and the PFS curve were intertwined at the beginning but began to separate between 3 to 6 months (eFigure in the Supplement).

**Figure 2.**
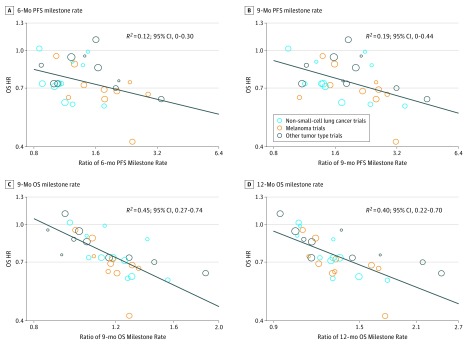
Correlation of Treatment Effects on the Overall Survival (OS) Hazard Ratio (HR) With the Ratios of OS or Progression-Free Survival (PFS) Milestone Rates A, Correlation of treatment effects on OS HR with the ratio of 6-month PFS milestone rate. B, Correlation of treatment effects on OS HR with the ratio of 9-month PFS milestone rate. C, Correlation of treatment effects on OS HR with the ratio of 9-month OS milestone rate. D, Correlation of treatment effects on OS HR with ratio of the 12-month OS milestone rate. Size of circles indicates size of trial (ie, larger circle indicates more participants).

Figure 2 shows the scatterplots of the treatment effects, illustrating trial-level correlations among the end points. The OS HR was weakly correlated with the ratio of the 6-month PFS milestone rate (*R*^2^ = 0.12; 95% CI, 0-0.30) (Figure 2A) and with the ratio of the 9-month PFS milestone rate (*R*^2^ = 0.19; 95% CI, 0-0.44) (Figure 2B). However, the OS HR was correlated with the ratio of the 9-month OS milestone rate (*R*^2^ = 0.45; 95% CI, 0.27-0.74) (Figure 2C) and the ratio of the 12-month OS milestone rate (*R*^2^ = 0.40; 95% CI, 0.22-0.70) (Figure 2D).

**Figure 3.**
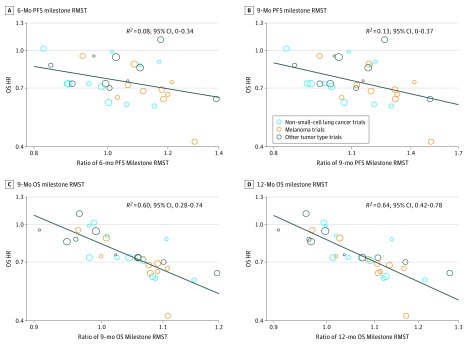
Correlation of Treatment Effects on Overall Survival (OS) Hazard Ratio (HR) With the Ratios of OS and Progression-Free Survival (PFS) Milestone Restricted Mean Survival Times (RMSTs) A, Correlation of treatment effects on OS HR with the ratio of 6-month PFS milestone RMST. B, Correlation of OS HR with the ratio of 9-month PFS milestone RMST. C, Correlation of treatment effects on OS HR with the ratio of 9-month OS milestone RMST. D, Correlation of treatment effects on OS HR with the ratio of 12-month OS milestone RMST. Size of circles indicates size of trial (ie, larger circle indicates more participants).

When the RMST was incorporated as a milestone treatment effect measurement, the OS HR correlated weakly with the 6-month PFS milestone RMST ratio (*R*^2^ = 0.08; 95% CI, 0-0.34) (Figure 3A) and with the 9-month PFS milestone RMST ratio (*R*^2^ = 0.13; 95% CI, 0-0.37) (Figure 3B). However, compared with the ratio of the 9-month or 12-month OS milestone rates, the ratio of the 9-month OS milestone RMST exhibited a strong correlation with the OS HR (*R*^2^ = 0.60; 95% CI, 0.28-0.74) (Figure 3C), and the correlation of the OS HR with the 12-month milestone RMST ratio was even stronger (*R*^2^ = 0.64; 95% CI, 0.42-0.78) (Figure 3D). In the leave-1-out cross-validation analysis, the median (range) *R*^2^ was 0.59 (0.57-0.64) for the 9-month OS milestone RMST and 0.64 (0.62-0.71) for the 12-month OS milestone RMST.

**Figure 4.**
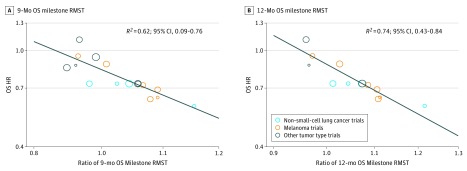
Correlation of Overall Survival (OS) Hazard Ratio (HR) With the Ratio of OS Milestone Restricted Mean Survival Times (RMSTs) Among Trials With Adequate Follow-up A, Correlation of treatment effects on OS HR with the ratio of 9-month OS milestone RMST among comparisons with a minimum follow-up longer than 9 months. B, Correlation of treatment effects on OS HR with the ratio of 12-month OS milestone RMST among comparisons with a minimum follow-up longer than 12 months. Size of circles indicates size of trial (ie, larger circle indicates more participants).

To provide a more reliable calculation of the OS milestone RMST, we assessed the correlation of the OS HR with the OS milestone RMST ratio by limiting the analysis to trials that set OS as the primary or coprimary end point and that had a minimum follow-up longer than 9 months (for the 9-month OS milestone RMST calculation) or 12 months (for the 12-month OS milestone RMST calculation). There were 16 comparisons that met criteria for the 9-month OS milestone RMST calculation and 13 comparisons that met the criteria for the 12-month OS milestone RMST calculation. In the 9-month comparison group, there was a slight improvement in the correlation of the OS HR with the ratio of the 9-month OS milestone RMST (*R*^2^ = 0.62; 95% CI, 0.09-0.76) (Figure 4A). Among the 12-month comparison group, the correlation of the OS HR with the ratio of the 12-month OS milestone RMST was significantly improved (*R*^2^ = 0.74; 95% CI, 0.43-0.84) (Figure 4B).

The proportional hazard assumption was violated in 10 of 31 comparisons (eTable 1 in the Supplement), which introduced uncertainty into the treatment effect measured by HR. To address this issue, we performed a sensitivity analysis in which comparisons with nonproportionality were excluded. In this analysis, a stronger correlation was observed of the OS HR with the ratios of the 9-month milestone RMST (*R*^2^ = 0.70; 95% CI, 0.38-0.90) or 12-month milestone RMST (*R*^2^ = 0.67; 95% CI, 0.39-0.87); however, there was no improvement in the correlation of the OS HR with the ratios of the 9-month milestone rate (*R*^2^ = 0.45; 95% CI 0.21-0.85) or 12-month milestone rate (*R*^2^ = 0.35; 95% CI 0.08-0.55). Moreover, the findings remained robust in additional sensitivity analyses in which the included trials were limited to trials with OS as the primary or coprimary end point, trials examining PDCD1 or PDCD1 ligand 1 inhibitors, phase 3 trials, trials examining only NSCLC, trials examining only melanoma, trials examining only cancers other than NSCLC and melanoma, first-line trials, and second-line or beyond trials (eTable 4 in the Supplement).

## Discussion

The results of this study demonstrate that the ratios of the OS milestone RMSTs had a stronger correlation with the OS HRs compared with the ratios of the OS milestone rates. In contrast, there was a poor correlation between the ratios of the PFS milestone rates or PFS milestone RMSTs and OS HRs. Notably, the correlations of the OS HR with the ratio of the OS milestone rate at 12 months or 9 months were much weaker than those reported in the study by Blumenthal et al^9^ (*R*^2^ = 0.45 vs *R*^2^ = 0.67 at 9 months and *R*^2^ = 0.40 vs *R*^2^ = 0.80 at 12 months). One possible explanation is that our analysis only included trials studying ICIs, whereas only 6 of the 25 included trials in the study by Blumenthal et al^9^ were ICI trials: 17% of the patients were treated with checkpoint inhibitor therapies, and the remainder were treated with chemotherapy and targeted therapies. Considering that these 3 therapeutic classes have different response patterns, the stronger correlations observed in the study by Blumenthal et al^9^ might reflect a mixed effect.

The ratio of the OS milestone rate at a given time point is unable to account for the survival information before that particular time point.^44^ Immune checkpoint inhibitors have delayed clinical benefits; therefore, it is likely that the ratio of the OS milestone rate may overestimate the treatment effect. The ratio of the 9-month OS milestone rate was even more closely correlated with the OS HR than the ratio of the 12-month OS milestone rate (*R*^2^ = 0.45 vs *R*^2^ = 0.40), which might have been caused by a more prominent overestimation of the treatment effect using the latter end point.

With the further introduction of the RMST for milestone treatment effect measurement, a substantial improvement was observed in the correlation of the OS HR with the ratio of the OS milestone RMST. Unfortunately, as the *R*^2^ did not reach the predefined validated cutoff value of 0.80, it may be too early to recommend the use of the OS milestone RMST as an intermediate end point for ICI trials. However, the results suggest that the OS milestone RMST outperforms the OS milestone rate as a potential intermediate end point for ICI trials and is worthy of further investigation. In addition, the OS milestone RMST may also be explored prospectively as a secondary end point in future ICI trials.

The use of the milestone RMST in the analyses not only improved the strength of the correlation with the OS HRs but also retained the advantage of milestone analysis, ie, the predictability and simplicity of the analysis as a time-driven end point. A mature time point is of great importance and should be predefined for milestone analysis, which is different from event-driven end points. Among trials with a minimum follow-up duration longer than 12 months, the correlation of the OS HR with the ratio of the 12-month OS milestone RMST was further improved, suggesting that data maturity is indispensable for the milestone RMST in terms of treatment effect measurement. Considering that future trials may increasingly use ICIs as controls in front-line design, in biomarker-enrichment strategies, and in combinational ICIs therapy,^9^ a substantially prolonged follow-up duration may be required to obtain the expected number of events. In contrast, the milestone RMST as a time-driven end point would have a well-defined maximum follow-up duration according to the prespecified milestone time point. Thus, while there should be a continuous optimization of the choice of milestones depending on increasing amounts of trial data, it is reasonable to further investigate the 12-month OS milestone RMST as an intermediate end point in future ICI trials. Moreover, even the 9-month OS milestone RMST ratio outperformed the ratio of the 12-month OS rate in terms of strength of correlation with OS HR, suggesting that OS milestone RMST ratio–based end points may require a shorter follow-up duration to detect early signals of the efficacy of ICI-based therapy.

The ratios of the PFS milestone rate and PFS milestone RMST showed weak correlation with OS HR. In most of the included trials, disease progression was defined using the standard RECIST version 1.1 criteria,^43^ which may not be able to fully capture the treatment effect of ICIs because of unique patterns of patient response.^45^ In addition, the correlation of PFS with OS was found to be weak in a previous study of 13 trials containing ICIs submitted to the US Food and Drug Administration, which suggests that PFS may not capture the survival benefit of patients with long-term survival.^5^ In 2018, Ritchie et al^6^ recommended the use of the 6-month PFS milestone rate as the preferred end point for phase 2 trials studying ICIs, primarily based on its correlation with the 12-month OS milestone rate. Our study found weak correlations of the ratio of 12-month OS milestone rate or 6-month PFS milestone rate with the OS HR, suggesting that it might be inappropriate to adopt the 6-month PFS rate as the primary end point in phase 2 ICI trials.

### Limitations

Several limitations should be acknowledged. Although the individual patient survival data were reconstructed accurately for milestone analysis, there was no access to detailed individual patient data, and information on patient-level associations with the examined end points could not be provided. In one trial,^23^ the difference between the reconstructed HR and the reported HR was greater than 5%, which may introduce potential bias to the findings. In addition, although only trials studying ICIs were included, the different study designs and patient populations among the included trials may have led to potential bias. The proportional hazard assumption was violated in some trials, which may have compromised the accuracy of HR as measurement of treatment effectiveness. Comparisons with nonproportionality were excluded and revealed correlations of the OS HR with the ratio of the 9-month or 12-month milestone RMST. Moreover, although assessment at the patient level is of value to identify appropriate surrogate end points, neither the milestone survival rate nor the milestone RMST are measurable at the individual patient level.

## Conclusions

In the present study, the results demonstrated that the ratios of the OS milestone RMST were more strongly correlated with the OS HRs than the ratios of the OS milestone rate, whereas there was weak correlation of OS HRs with the ratios of the PFS milestone rates and with the PFS milestone RMSTs. Although the OS milestone RMST should not yet be used as an intermediate end point for future ICI trials, it could be prospectively incorporated as a secondary end point for further testing in future studies with front-line strategies involving ICI combinations or biomarker-enrichment designs. To validate the OS milestone RMST as an intermediate end point for ICI trials and to optimize the choice of milestone time points, further studies are required.

## Notes

Supplement. **eMethods.** Search Strategy **eTable 1.** Detailed Regimen and Characteristics of Trials Included **eTable 2.** Comparison of Reported Overall Survival Results With Those Calculated Based on Reconstructed Individual Patient Data **eTable 3.** Comparison of Reported Progression-Free Survival Results With Those Calculated Based on Reconstructed Individual Patient Data **eTable 4.** Sensitivity Analyses in Which A, Only Trials With Primary or Coprimary End Point of Overall Survival Were Included; B, Only PDCD1 or PDCD1 Ligand 1 Inhibitors Trials Were Included; C, Only Phase 3 Trials Were Included; D, Only NSCLC Trials Were Included; E, Only Melanoma Trials Were Included; F, Only Trials Other Than NSCLC and Melanoma Were Included; G, Only First-Line Trials Were Included; and H, Only Second-Line or Beyond Trials Were Included **eFigure.** Kaplan-Meier Survival Estimates **eReferences**
