# Supplementary material for: Correlation of Milestone Restricted Mean Survival Time Ratio With Overall Survival Hazard Ratio in Randomized Clinical Trials of Immune Checkpoint Inhibitors: A Systematic Review and Meta-analysis
Source: JAMA Netw Open. 2019 May 3;2(5):e193433. doi: 10.1001/jamanetworkopen.2019.3433 (PMC6503508; doi:10.1001/jamanetworkopen.2019.3433)
Supplement: Supplement. — eMethods. Search Strategy eTable 1. Detailed Regimen and Characteristics of Trials Included eTable 2. Comparison of Reported Overall Survival Results With Those Calculated Based on Reconstructed Individual Patient Data eTable 3. Comparison of Reported Progression-Free Survival Results With Those Calculated Based on Reconstructed Individual Patient Data eTable 4. Sensitivity Analyses in Which A, Only Trials With Primary or Coprimary End Point of Overall Survival Were Included; B, Only PDCD1 or PDCD1 Ligand 1 Inhibitors Trials Were Included; C, Only Phase 3 Trials Were Included; D, Only NSCLC Trials Were Included; E, Only Melanoma Trials Were Included; F, Only Trials Other Than NSCLC and Melanoma Were Included; G, Only First-Line Trials Were Included; and H, Only Second-Line or Beyond Trials Were Included eFigure. Kaplan-Meier Survival Estimates eReferences [file jamanetwopen-2-e193433-s001.pdf]

Supplementary Online Content

Wang Z-X, Wu H-X, Xie L, et al. Correlation of milestone restricted mean survival time ratio with overall survival hazard ratio in randomized clinical trials of immune checkpoint inhibitors: a systematic review and meta-analysis. *JAMA Netw Open*. 2019;2(5):e193433. doi:10.1001/jamanetworkopen.2019.3433

eMethods. Search Strategy.
eTable 1. Detailed Regimen and Characteristics of Trials Included
eTable 2. Comparison of Reported Overall Survival Results With Those Calculated Based on Reconstructed Individual Patient Data
eTable 3. Comparison of Reported Progression-Free Survival Results With Those Calculated Based on Reconstructed Individual Patient Data
eTable 4. Sensitivity Analyses in Which A, Only Trials With Primary or Coprimary End Point of Overall Survival Were Included; B, Only PDCD1 or PDCD1 Ligand 1 Inhibitors Trials Were Included; C, Only Phase 3 Trials Were Included; D, Only NSCLC Trials Were Included; E, Only Melanoma Trials Were Included; F, Only Trials Other Than NSCLC and Melanoma Were Included; G, Only First-Line Trials Were Included; and H, Only Second-Line or Beyond Trials Were Included
eFigure. Kaplan-Meier Survival Estimates
eReferences

This supplementary material has been provided by the authors to give readers additional information about their work.

© 2019 Wang Z-X et al. *JAMA Network Open.*

eMethods

Search Strategy

A. Medline (PubMed)

#1 randomized controlled trial [pt]

#2 controlled clinical trial [pt]

#3 randomized [tiab]

#4 placebo [tiab]

#5 clinical trials as topic [mesh: noexp]

#6 randomly [tiab]

#7 trial [ti]

#8 #1 OR #2 OR #3 OR #4 OR #5 OR #6 OR #7

#9 animals [mh] NOT humans [mh]

#10 #8 NOT #9

#11 Nivolumab

#12 Opdivo

#13 BMS-936558

#14 MDX1106

#15 ONO-4538

#16 BMS-936559

#17 MDX1105

#18 Pembrolizumab

#19 Lambrolizumab

#20 Keytruda

#21 MK-3475

#22 Atezolizumab

#23 MPDL3280

#24 MPDL3280A

#25 RG7446

#26 Tecentriq

#27 Avelumab

#28 MSB0010718C

#29 Durvalumab

#30 MEDI4736

#31 cemiplimab

#32 REGN2810

#33 PD-1 inhibitor

#34 Programmed death 1 inhibitor

#35 Anti-PD-1

#36 Anti-Programmed Cell Death 1

#37 PD-L1 inhibitor

#38 Programmed death ligand 1 inhibitor

#39 Anti-PD-L1

#40 Anti-Programmed Cell Death Ligand-1

#41 Checkpoint Inhibitor

© 2019 Wang Z-X et al. *JAMA Network Open.*

#42 Checkpoint blockade
#43 Programmed Cell Death 1 Receptor [mesh]
#44 CTLA4 protein, human
#45 anti CTLA4
#46 anti CTLA-4
#47 cytotoxic T-lymphocyte-associated antigen 4
#48 Cytotoxic T lymphocyte antigen 4
#49 CTLA-4
#50 Cytotoxic T-lymphocyte protein 4
#51 anti-CTLA4 antibodies
#52 Ipilimumab
#53 MDX-010
#54 MDX-101
#55 BMS-734016
#56 Yervoy
#57 Antigens, CD/immunology* [mesh]
#58 CTLA-4 Antigen [mesh]
#59 #11 or #12 or #13 or #14 or #15 or #16 or #17 or #18 or #19 or #20 or #21 or #22 or #23 or #24 or #25 or #26 or #27 or #28 or #29 or #30 or #31 or #32 or #33 or #34 or #35 or #36 or #37 or #38 or #39 or #40 or #41 or #42 or #43 or #44 or #45 or #46 or #47 or #48 or #49 or #50 or #51 or #52 or #53 or #54 or #55 or #56 or #57 or #58
#60 #10 and #59

B. Embase (Ovid)

1 Nivolumab
2 Opdivo
3 BMS-936558
4 MDX1106
5 ONO-4538
6 BMS-936559
7 MDX 1105
8 Pembrolizumab
9 Lambrolizumab
10 Keytruda
11 MK-3475
12 Atezolizumab
13 MPDL3280
14 MPDL3280A
15 RG7446
16 Tecentriq
17 Avelumab
18 MSB0010718C
19 Durvalumab
20 MEDI4736
21 cemiplimab

© 2019 Wang Z-X et al. *JAMA Network Open.*

22 REGN2810
23 PD-1 inhibitor
24 Programmed death 1 inhibitor
25 Anti-PD-1
26 Anti-Programmed Cell Death 1
27 PD-L1 inhibitor
28 Programmed death ligand 1 inhibitor
29 Anti-PD-L1
30 Anti-Programmed Cell Death Ligand-1
31 Checkpoint Inhibitor
32 Checkpoint blockade
33 Programmed Cell Death 1 Receptor
34 CTLA4 protein, human
35 anti CTLA4
36 anti CTLA-4
37 cytotoxic T-lymphocyte-associated antigen 4
38 Cytotoxic T lymphocyte antigen 4
39 CTLA-4
40 Cytotoxic T-lymphocyte protein 4
41 anti-CTLA4 antibodies
42 Antigens, CD4
43 MDX-010
44 MDX-101
45 BMS-734016
46 Yervoy
47 CTLA-4 Antigen
48 Ipilimumab
49 1 or 2 or 3 or 4 or 5 or 6 or 7 or 8 or 9 or 10 or 11 or 12 or 13 or 14 or 15 or 16 or 17 or 18 or 19 or 20 or 21 or 22 or 23 or 24 or 25 or 26 or 27 or 28 or 29 or 30 or 31 or 32 or 33 or 34 or 35 or 36 or 37 or 38 or 39 or 40 or 41 or 42 or 43 or 44 or 45 or 46 or 47 or 48
50 'crossover procedure':de OR 'double-blind procedure':de OR 'randomized controlled trial':de OR 'singleblind procedure':de OR (random* OR factorial* OR crossover* OR cross NEXT/1 over* OR placebo* OR doubl* NEAR/1 blind* OR singl* NEAR/1 blind* OR assign* OR allocat* OR volunteer*):de,ab,ti
51 49 and 50

C. Cochrane Central Register of Controlled Trials

#1 Nivolumab
#2 Opdivo
#3 BMS-936558
#4 MDX1106
#5 ONO-4538
#6 BMS-936559
#7 MDX 1105
#8 Pembrolizumab

© 2019 Wang Z-X et al. *JAMA Network Open.*

#9 Lambrolizumab
#10 Keytruda
#11 MK-3475
#12 Atezolizumab
#13 MPDL3280
#14 MPDL3280A
#15 RG7446
#16 Tecentriq
#17 Avelumab
#18 MSB0010718C
#19 Durvalumab
#20 MEDI4736
#21 cemiplimab
#22 REGN2810
#23 PD-1 inhibitor
#24 Programmed death 1 inhibitor
#25 Anti-PD-1
#26 Anti-Programmed Cell Death 1
#27 PD-L1 inhibitor
#28 Programmed death ligand 1 inhibitor
#29 Anti-PD-L1
#30 Anti-Programmed Cell Death Ligand-1
#31 Checkpoint Inhibitor
#32 Checkpoint blockade
#33 MeSH descriptor: [Programmed Cell Death 1 Receptor] explode all trees
#34 CTLA4 protein, human
#35 anti CTLA4
#36 anti CTLA-4
#37 cytotoxic T-lymphocyte-associated antigen 4
#38 Cytotoxic T lymphocyte antigen 4
#39 CTLA-4
#40 Cytotoxic T-lymphocyte protein 4
#41 anti-CTLA4 antibodies
#42 Antigens, CD
#43 MDX-010
#44 MDX-101
#45 BMS-734016
#46 Yervoy
#47 CTLA-4 Antigen
#48 Ipilimumab.mp
#49 MeSH descriptor: [Antigens, CD4] explode all trees
#50 MeSH descriptor: [CTLA-4 Antigens] explode all trees
#51 #1 or #2 or #3 or #4 or #5 or #6 or #7 or #8 or #9 or #10 or #11 or #12 or #13 or #14 or #15 or #16 or #17 or #18 or #19 or #20 or #21 or #22 or #23 or #24 or #25 or #26 or #27 or #28 or #29 or #30

© 2019 Wang Z-X et al. *JAMA Network Open.*

or #31 or #32 or #33 or #34 or #35 or #36 or #37 or #38 or #39 or #40 or #41 or #42 or #43 or #44 or #45 or #46 or #47 or #48 or #49 or #50

© 2019 Wang Z-X et al. *JAMA Network Open.*

eTable 1. Detailed Regimen and Characteristics of Trials Included

Trial	Experimental arm	Control arm	Immune target	Tumor stage	Age (years)[a]		P^b for proportional hazards test
					Experimental	Control	
Robert et al,[1] 2015	Nivolumab 3mg/kg q2w	Dacarbazine 1000mg/m^2 q3w	PD-1	unresectable stage III or IV	64 (18-86)	66 (26-87)	**0.006**
Hodi et al,[2] 2016	(Nivolumab 1mg/kg q3w + Ipilimumab 10 mg/kg q3w) x 4 then Nivolumab 3mg/kg q2w	(Placebo 1mg/kg q3w + Ipilimumab 10 mg/kg q3w) x 4 then Placebo 3mg/kg q2w	PD-1 + CTLA-4	unresectable stage III or IV	64 (27-87)	67 (31-80)	0.270
Larkin et al,[3] 2018	Nivolumab 3mg/kg q2w	Physician's choice chemotherapy	PD-1	unresectable stage IIIC or IV metastatic	59 (23-88)	62 (29-85)	0.116
Robert et al,[4] 2015	A: Pembrolizumab 10mg/kg q2w<br>B: Pembrolizumab 10mg/kg q3w	Ipilimumab 3mg/kg q3w	PD-1	unresectable stage III or IV	63 (22-89)	62 (18-88)	0.404<br>0.153
Hodi et al,[5] 2010	A: Ipilimumab 3mg/kg q3w + glycoprotein 100 q3w<br>B: Ipilimumab 3mg/kg q3w + placebo q3w	Glycoprotein 100 q3w	CTLA-4	unresectable stage III or IV	55.6 (NA)<br>56.8 (NA)	57.4 (NA)	0.538<br>0.355
Robert et al,[6] 2011	Ipilimumab 10mg/kg q3w × 4 and maintenance q12w + dacarbazine 850mg/m^2 q3w ×8	Dacarbazine 850mg/m^2 q3w ×8	CTLA-4	unresectable stage III or IV	57.5 (NA)	56.4 (NA)	0.954
Hodi et al,[7] 2014	Ipilimumab 10mg/kg q3w + sargramostim 250μg D1-14 q3w	Ipilimumab 10mg/kg q3w	CTLA-4	unresectable stage III or IV	61 (25-86)	64 (21-89)	0.452
Ribas et al,[8] 2013	Tremelimumab 15mg/kg q90d	Physician's choice chemotherapy	CTLA-4	unresectable stage IIIc or IV	57 (22-90)	56 (22-90)	0.561

© 2019 Wang Z-X et al. *JAMA Network Open.*

Fehrenbacher et al,[9] 2016	Atezolizumab 1200mg q3w	Docetaxel 75mg/m^2 q3w	PD-L1	advanced or metastastic	62 (42-82)	62 (36-84)	**0.099**
Rittmeyer et al,[10] 2017	Atezolizumab 1200mg q3w	Docetaxel 75mg/m^2 q3w	PD-L1	stage IIIB or IV	63 (33-82)	64 (34-85)	0.442
Borghaei et al,[11] 2015	Nivolumab 3mg/kg q2w	Docetaxel 75mg/m^2 q3w	PD-1	stage IIIB/IV or recurrent	61 (37-84)	64 (21-85)	**0.002**
Brahmer et al,[12] 2015	Nivolumab 3 mg/kg q2w	Docetaxel 75mg/m^2 q3w	PD-1	stage IIIB/IV	62 (39-85)	64 (42-84)	0.439
Carbone et al,[13] 2017	Nivolumab 3mg/kg q2w	Physician's choice chemotherapy	PD-1	stage IV or recurrent	63 (33-89)	65 (29-87)	0.479
Herbst et al,[14] 2016	A: Pembrolizumab 2mg/kg q3w<br>B: Pembrolizumab 10mg/kg q3w	Docetaxel 75mg/m^2 q3w	PD-1	advanced	63 (56-69)<br>63 (56–69)	62 (56-69)	0.318<br>**0.066**
Langer et al,[15] 2016	(Pembrolizumab 200mg + Pemetrexed 500mg/m^2 + Carboplatin AUC5) q3w x 4 + maintenance pembrolizumab up to 24 months +/- maintenance pemetrexed	(Pemetrexed 500mg/m^2 + Carboplatin AUC5) q3w x 4 +/- maintenance pemetrexed	PD-1	stage IIIB or IV	62·5 (54-70)	63·2 (58-70)	0.608
Reck et al,[16] 2016	Pembrolizumab 200mg q3w	Physician's choice chemotherapy	PD-1	stage IV	64.5 (33-90)	66.0 (38-85)	0.944
Lynch et al,[17] 2012	A: Concurrent Ipilimumab 10 mg/kg q3w × 4 + carboplatin AUC6 + paclitaxel 175mg/m^2 ×6<br>B: Phased ipilimumab 10 mg/kg q3w ×4 + carboplatin AUC6 + paclitaxel 175mg/m^2 ×6	Carboplatin AUC6 + paclitaxel 175 mg/m^2	CTLA-4	stage IIIB/IV or recurrent	61 (36-88)<br>59 (36-82)	62 (36-82)	0.766<br>0.111

© 2019 Wang Z-X et al. *JAMA Network Open.*

Kang et al,[18] 2017	Nivolumab 3mg/kg q2w	Placebo	PD-1	unresectable advanced or recurrent	62 (54-69)	61 (53-68)	0.405
Ferris et al,[19] 2016	Nivolumab 3mg/kg q2w	Physician's choice chemotherapy	PD-1	recurrent or metastastic	59 (29-83)	61 (28-78)	**0.004**
Kwon et al,[20] 2014	Radiotherapy 8Gy + ipilimumab 10mg/kg q3w	Radiotherapy 8Gy + placebo 10mg/kg q3w	CTLA-4	advanced	69 (47-86)	67.5 (45-86)	**<0.001**
Beer et al,[21] 2017	Ipilimumab 10mg/kg q3w x 4 + maintenance 10mg/kg q12w	Placebo	CTLA-4	metastatic	70 (44-91)	69 (42-92)	**0.089**
Motzer et al,[22] 2015	Nivolumab 3mg/kg q2w	Everolimus 10mg daily	PD-1	advanced or metastatic	62 (23-88)	62 (18-86)	0.121
Reck et al,[23] 2013	A: Concurrent ipilimumab 10mg/kg q3w × 6 and maintenance q12w + carboplatin AUC6 + paclitaxel 175mg/m^2<br>B: Phased ipilimumab 10mg/kg q3w × 6 and maintenance q12w + carboplatin AUC6 + paclitaxel 175mg/m^2	Carboplatin AUC6 + paclitaxel 175 mg/m^2	CTLA-4	extensive-stage	59 (43-80)<br>57 (44-80)	58 (42-82)	**0.076**<br>0.811
Reck et al,[24] 2016	Ipilimumab 10mg/kg q3w + cisplatin 75mg/m^2 or carboplatin AUC5 D1 + etoposide 100mg/m^2 D1-3	Placebo + cisplatin 75mg/m^2 or carboplatin AUC5 D1 + etoposide 100mg/m^2 D1-3	CTLA-4	extensive-stage	62 (39-85)	63 (36-81)	0.248
Powels et al,[25] 2018	Atezolizumab 1200mg q3w	Physician's choice chemotherapy	PD-L1	metastatic	67 (43-88)	67 (36-84)	**0.014**
Bellmunt et al,[26] 2017	Pembrolizumab 200mg q3w	Physician's choice chemotherapy	PD-1	advanced	67 (29-88)	65 (26-84)	**0.036**

© 2019 Wang Z-X et al. *JAMA Network Open.*

Abbreviation: mg/kg, milligram per kilogram; mg/m^2, milligram per square metre; q2w, once every 2 weeks; q3w, once every 3 weeks; q12w, once every 12 weeks; q90d, once every 90 days; AUC, area under the curve.

a. Median (range)

b. *P* value in bold denotes significant violation of proportional hazard assumption.

© 2019 Wang Z-X et al. *JAMA Network Open.*

eTable 2. Comparison of Reported Overall Survival Results With Those Calculated Based on Reconstructed Individual Patient Data.

Trial	HR				Median OS in the treatment group (months)				Median OS in the control group (months)			
	Reported	Calculated	Absolute Difference	Relative Difference	Reported	Calculated	Absolute Difference	Relative Difference	Reported	Calculated	Absolute Difference	Relative Difference
Robert et al,[1] 2015	0.42	0.40	-0.02	-4.3%	NR	NR	NA	NA	10.8	10.9	0.1	0.9%
Hodi et al,[2] 2016	0.74	0.74	0.00	-0.5%	NR	NR	NA	NA	NR	NR	NA	NA
Larkin et al,[3] 2018	0.95	0.92	-0.03	-3.0%	15.7	15.8	0.1	0.6%	14.4	14.5	0.1	0.7%
Robert et al,[4] 2015[a]	0.69	0.66	-0.03	-4.1%	NR	NR	NA	NA	NR	NR	NA	NA
Robert et al,[4] 2015[b]	0.63	0.63	0.00	0%	NR	NR	NA	NA	NR	NR	NA	NA
Hodi et al[5], 2010[c]	0.68	0.71	0.03	3.8%	10	10.14	0.14	1.4%	6.4	6.47	0.07	1.1%
Hodi et al,[5] 2010[d]	0.66	0.64	-0.02	-2.5%	10.1	10.2	0.1	1.0%	6.4	6.47	0.07	1.1%
Robert et al,[6] 2011	0.72	0.76	0.04	5.9%	11.2	11.26	0.06	0.5%	9.1	9.13	0.03	0.3%
Hodi et al,[7] 2014	0.64	0.67	0.03	4.2%	17.5	17.7	0.2	1.1%	12.7	12.5	-0.2	-1.6%
Ribas et al,[8] 2013	0.88	0.87	-0.01	-1.2%	12.6	12.6	0	0.0%	10.7	10.8	0.1	0.9%
Fehrenbacher et al,[9] 2016	0.73	0.73	0.00	0.5%	12.6	12.5	-0.1	-0.8%	9.7	9.85	0.15	1.5%
Rittmeyer et al,[10] 2017	0.73	0.74	0.01	0.9%	13.8	13.71	-0.09	-0.7%	9.6	9.64	0.04	0.4%
Borghaei et al,[11] 2015	0.73	0.76	0.03	4.4%	12.2	12.27	0.07	0.6%	9.4	9.41	0.01	0.1%
Brahmer et al,[12] 2015	0.59	0.59	0.00	-0.2%	9.2	9.31	0.11	1.2%	6	6.07	0.07	1.2%
Carbone et al,[13] 2017	1.02	1.05	0.03	2.6%	14.4	14.5	0.1	0.7%	13.2	13.3	0.1	0.8%
Herbst et al,[14] 2016[e]	0.61	0.63	0.02	3.8%	12.7	12.64	-0.06	-0.5%	8.5	8.57	0.07	0.8%
Herbst et al,[14] 2016[f]	0.71	0.73	0.02	3.1%	10.4	10.45	0.05	0.5%	8.5	8.57	0.07	0.8%
Langer et al,[15] 2016	0.9	0.94	0.04	4.6%	NR	NR	NA	NA	NR	NR	NA	NA
Reck et al,[16] 2016	0.6	0.61	0.01	2.5%	NR	NR	NA	NA	NR	NR	NA	NA
Lynch et al,[17] 2012[g]	0.87	0.86	-0.01	-1.2%	12.2	12.19	-0.01	-0.1%	8.3	8.27	-0.03	-0.4%

© 2019 Wang Z-X et al. *JAMA Network Open.*

Lynch et al,[17] 2012[h]	0.99	0.99	0.00	0.1%	9.69	9.76	0.07	0.7%	8.28	8.27	-0.01	-0.1%
Kang et al,[18] 2017	0.63	0.64	0.01	2.3%	5.26	5.32	0.06	1.1%	4.14	4.21	0.07	1.7%
Ferris et al,[19] 2016	0.7	0.71	0.01	1.3%	7.5	7.56	0.06	0.8%	5.1	5.1	0	0.0%
Kwon et al,[20] 2014	0.85	0.85	0.00	0.1%	11.2	11.2	0	0.0%	10	10.1	0.1	1.0%
Beer et al,[21] 2017	1.11	1.07	-0.04	-3.8%	28.7	28.4	-0.3	-1.0%	29.7	29.6	-0.1	-0.3%
Motzer et al,[22] 2015	0.73	0.76	0.03	3.5%	25	24.9	-0.1	-0.4%	19.6	19.5	-0.1	-0.5%
Reck et al,[23] 2013[i]	0.75	0.77	0.02	2.6%	12.9	13.01	0.11	0.9%	9.9	9.93	0.03	0.3%
Reck et al,[23] 2013[j]	0.95	0.95	0.00	-0.3%	9.13	9.12	-0.01	-0.1%	9.92	9.93	0.01	0.1%
Reck et al,[24] 2016	0.94	0.93	-0.01	-0.7%	11	11.1	0.1	0.9%	10.9	10.8	-0.1	-0.9%
Powels et al,[25] 2018	0.87	0.83	-0.04	-4.8%	11.1	11.1	0	0.0%	10.6	10.8	0.2	1.9%
Bellmunt et al,[26] 2017	0.73	0.73	0.00	-0.5%	10.3	10.49	0.19	1.8%	7.4	7.35	-0.05	-0.7%

Abbreviations: OS, overall survival; HR, hazard ratio; NR, not reached; NA, not avaliable.

a. Pembrolizumab q3w vs. ipilimumab.

b. Pembrolizumab q2w vs. ipilimumab.

c. Ipilimumab + glycoprotein 100 vs. glycoprotein 100.

d. Ipilimumab vs. glycoprotein 100.

e. Pembrolizumab 10mg/kg vs. Docetaxel.

f. Pembrolizumab 2mg/kg vs. Docetaxel.

g. Phased ipilimumab + carboplatin + paclitaxel vs. carboplatin + paclitaxel.

h. Concurrent ipilimumab + carboplatin + paclitaxel vs. carboplatin + paclitaxel.

i. Phased ipilimumab + carboplatin + paclitaxel vs. carboplatin + paclitaxel.

j. Concurrent ipilimumab + carboplatin + paclitaxel vs. carboplatin + paclitaxel.

© 2019 Wang Z-X et al. *JAMA Network Open.*

eTable 3. Comparison of Reported Progression-Free Survival Results With Those Calculated Based on Reconstructed Individual Patient Data

Trial	HR				Median PFS in the treatment group (months)				Median PFS in the control group (months)			
	Reported	Calculated	Absolute Difference	Relative Difference	Reported	Calculated	Absolute Difference	Relative Difference	Reported	Calculated	Absolute Difference	Relative Difference
Robert et al,[1] 2015	0.43	0.47	0.04	9.0%	5.1	5.21	0.11	2.2%	2.2	2.21	0.01	0.5%
Hodi et al,[2] 2016	0.36	0.37	0.01	2.1%	NR	NA	NA	NA	3	3.17	0.17	5.7%
Larkin et al,[3] 2018	1.03	1.00	-0.03	-3.1%	3.1	3	-0.1	-3.2%	3.7	3.79	0.09	2.4%
Robert et al,[4] 2015[a]	0.58	0.59	0.01	1.4%	4.1	4.11	0.01	0.2%	2.8	2.82	0.02	0.7%
Robert et al,[4] 2015[b]	0.58	0.62	0.04	6.3%	5.5	5.7	0.2	3.6%	2.8	2.82	0.02	0.7%
Hodi et al[5], 2010[c]	0.81	0.82	0.01	1.7%	2.76	2.78	0.02	0.7%	2.76	2.76	0	0.0%
Hodi et al,[5] 2010[d]	0.64	0.69	0.05	7.2%	2.86	2.81	-0.05	-1.7%	2.76	2.76	0	0.0%
Robert et al,[6] 2011	0.76	0.78	0.02	2.5%	NA	2.76	NA	NA	NA	2.6	NA	NA
Hodi et al,[7] 2014	0.87	0.92	0.05	5.8%	3.1	3.08	-0.02	-0.6%	3.1	3.16	0.06	1.9%
Ribas et al,[8] 2013	NA	0.27	NA	NA	35.8	35.8	0	0.0%	13.7	13.7	0	0.0%
Fehrenbacher et al,[9] 2017	0.94	0.93	-0.01	-1.1%	2.7	2.71	0.01	0.4%	3	3.22	0.22	7.3%
Rittmeyer et al,[10] 2017	0.95	0.95	0.00	-0.2%	2.8	2.68	-0.12	-4.3%	4	3.98	-0.02	-0.5%
Borghaei et al,[11] 2015	0.92	0.91	-0.01	-1.4%	2.3	2.39	0.09	3.9%	4.2	4.29	0.09	2.1%
Brahmer et al,[12] 2015	0.62	0.61	-0.01	-1.1%	3.5	3.58	0.08	2.3%	2.8	2.87	0.07	2.5%
Carbone et al,[13] 2017	1.15	1.17	0.02	1.6%	4.2	4.3	0.1	2.4%	5.9	5.95	0.05	0.8%
Herbst et al,[14] 2016[e]	0.79	0.78	-0.01	-1.9%	4	4.03	0.03	0.8%	4	4.07	0.07	1.8%
Herbst et al,[14] 2016[f]	0.88	0.87	-0.01	-0.6%	3.9	3.87	-0.03	-0.8%	4	4.07	0.07	1.8%
Langer et al,[15] 2016	0.53	0.53	0.00	0.6%	13	13	0	0.0%	8.9	8.9	0	0.0%
Reck et al,[16] 2016	0.5	0.49	-0.01	-2.3%	10.3	10.36	0.06	0.6%	6	6.08	0.08	1.3%

© 2019 Wang Z-X et al. *JAMA Network Open.*

Lynch et al,[17] 2012[g]	0.72	0.72	0.00	0.1%	5.68	5.71	0.03	0.5%	4.63	4.68	0.05	1.1%
Lynch et al,[17] 2012[h]	0.81	0.80	-0.01	-1.6%	5.52	5.55	0.03	0.5%	4.63	4.68	0.05	1.1%
Kang et al,[18] 2017	0.6	0.62	0.02	3.8%	1.61	1.63	0.02	1.2%	1.45	1.48	0.03	2.1%
Ferris et al,[19] 2016	0.89	0.89	0.00	-0.5%	2	2.07	0.07	3.5%	2.3	2.38	0.08	3.5%
Kwon et al,[20] 2014	0.7	0.71	0.01	1.6%	4	4.01	0.01	0.2%	3.1	3.13	0.03	1.0%
Beer et al,[21] 2016	0.67	0.68	0.01	1.4%	5.6	5.47	-0.13	-2.3%	3.8	3.81	0.01	0.3%
Motzer et al,[22] 2015	0.88	0.86	-0.02	-1.9%	4.6	4.55	-0.05	-1.1%	4.4	4.52	0.12	2.7%
Reck et al,[23] 2013[i]	0.64	0.62	-0.02	-3.3%	6.44	6.43	-0.01	-0.2%	5.26	5.27	0.01	0.2%
Reck et al,[23] 2013[j]	0.75	0.76	0.01	1.7%	5.68	5.6	-0.08	-1.4%	5.26	5.27	0.01	0.2%
Reck et al,[24] 2016	0.85	0.87	0.02	2.7%	4.6	4.56	-0.04	-0.9%	4.4	4.36	-0.04	-0.9%
Powels et al,[25] 2018	1.01	1.00	-0.01	-0.9%	2.4	2.23	-0.17	-7.1%	4.2	4.39	0.19	4.5%
Bellmunt et al,[26] 2017	0.98	0.94	-0.04	-4.0%	2.1	2.11	0.01	0.5%	3.3	3.24	-0.06	-1.8%

Abbreviations: PFS, progression-free survival; HR, hazard ratio; NR, not reached; NA, not avaliable.

a. Pembrolizumab q3w vs. ipilimumab.
b. Pembrolizumab q2w vs. ipilimumab.
c. Ipilimumab + glycoprotein 100 vs. glycoprotein 100.
d. Ipilimumab vs. glycoprotein 100.
e. Pembrolizumab 10mg/kg vs. Docetaxel.
f. Pembrolizumab 2mg/kg vs. Docetaxel.
g. Phased ipilimumab + carboplatin + paclitaxel vs. carboplatin + paclitaxel.
h. Concurrent ipilimumab + carboplatin + paclitaxel vs. carboplatin + paclitaxel.
i. Phased ipilimumab + carboplatin + paclitaxel vs. carboplatin + paclitaxel.
j. Concurrent ipilimumab + carboplatin + paclitaxel vs. carboplatin + paclitaxel.

© 2019 Wang Z-X et al. *JAMA Network Open.*

eTable 4. Sensitivity Analyses in Which A, Only Trials with Primary or Coprimary End Point of Overall Survival Were Included; B, Only Programmed Cell Death 1 or Programmed Cell Death 1 Ligand 1 Inhibitors Trials Were Included; C, Only Phase 3 Trials Were Included; D, Only Non–Small Cell Lung Cancer Trials Were Included; E, Only Melanoma Trials Were Included; F, Only Trials Other Than Non–Small Cell Lung Cancer and Melanoma Were Included; G, Only First-Line Trials Were Included; and H, Only Second-Line or Beyond Trials Were Included

Sensitivity analyses	R^2 (95% confidence interval)			
	**A**	**B**	**C**	**D**
OS HR with ratio of 9-month OS rate	0.46 (0.15-0.67)	0.54 (0.22-0.80)	0.50 (0.11-0.72)	0.54 (0.05-0.92)
OS HR with ratio of 12-month OS rate	0.41 (0.18-0.72)	0.44 (0.05-0.82)	0.41 (0.17-0.71)	0.64 (0.12-0.85)
OS HR with ratio of 9-month OS RMST	0.61 (0.29-0.75)	0.58 (0.23-0.70)	0.62 (0.24-0.73)	0.65 (0.01-0.95)
OS HR with ratio of 12-month OS RMST	0.66 (0.41-0.78)	0.66 (0.35-0.80)	0.67 (0.38-0.79)	0.68 (0.01-0.94)
	R^2 (95% confidence interval)			
	**E**	**F**	**G**	**H**
OS HR with ratio of 9-month OS rate	0.64 (0.20-0.96)	0.60 (0.03-0.86)	0.70 (0.37-0.98)	0.49 (0.12-0.69)
OS HR with ratio of 12-month OS rate	0.56 (0.17-0.91)	0.52 (0.01-0.80)	0.73 (0.48-0.94)	0.36 (0.00-0.57)
OS HR with ratio of 9-month OS RMST	0.77 (0.46-0.99)	0.61 (0.00-0.83)	0.80 (0.60-0.98)	0.70 (0.20-0.82)
OS HR with ratio of 12-month OS RMST	0.82 (0.56-0.99)	0.67 (0.00-0.88)	0.87 (0.66-0.99)	0.69 (0.37-0.83)

Abbreviations: HR, hazard ratio; RMST, restricted mean survival time.

© 2019 Wang Z-X et al. *JAMA Network Open.*

eFigure. Kaplan-Meier Survival Estimates.

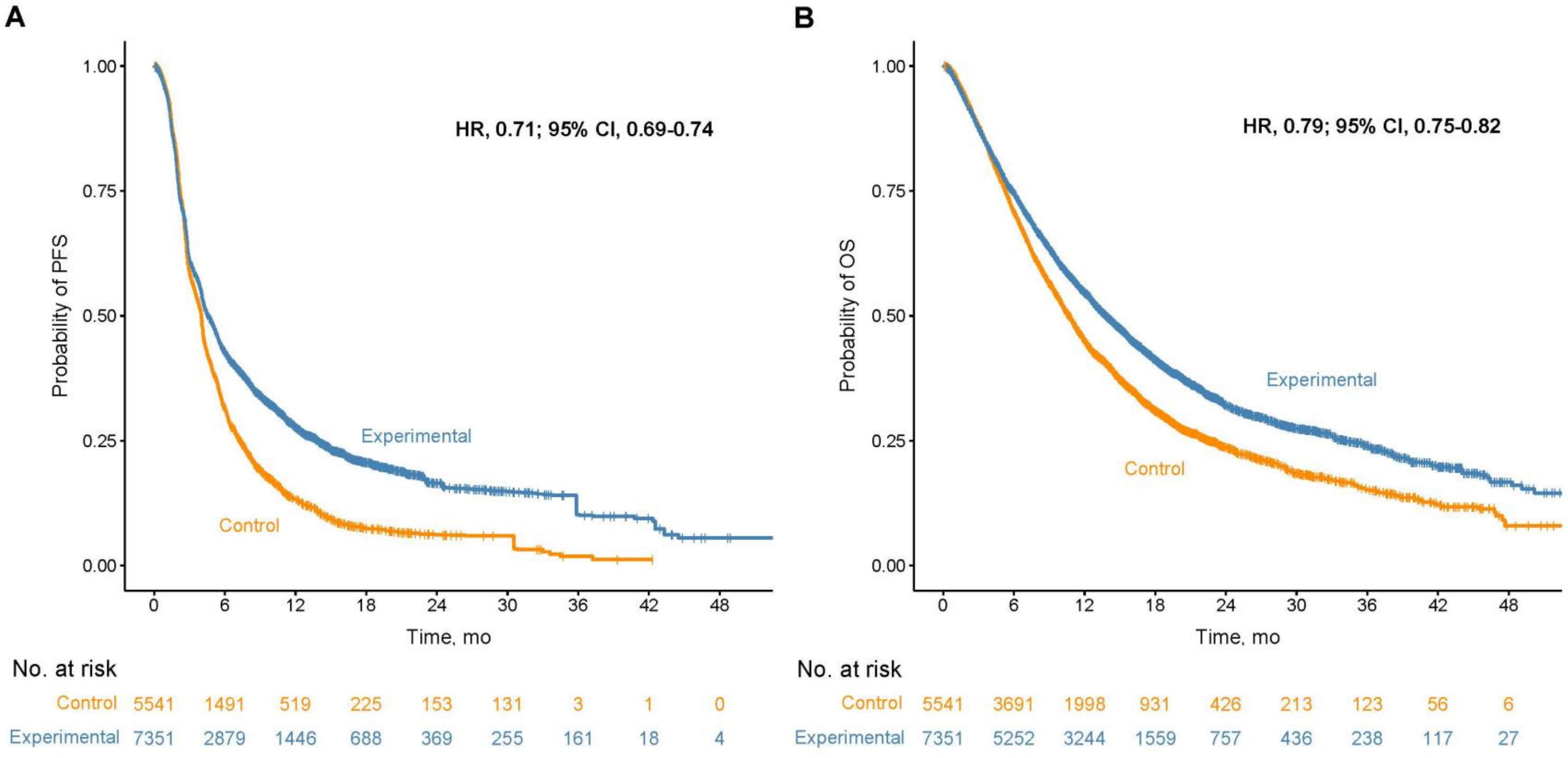

Progression free survival (A) and overall survival (B) were compared between experimental and control groups by pooling of the total 12892 patients. HR, hazard ratio; | , censored.

© 2019 Wang Z-X et al. *JAMA Network Open.*

eReferences

1. Robert C, Long GV, Brady B, et al. Nivolumab in previously untreated melanoma without BRAF mutation. *N Engl J Med.* 2015;372(4):320-330.
2. Hodi FS, Chesney J, Pavlick AC, et al. Combined nivolumab and ipilimumab versus ipilimumab alone in patients with advanced melanoma: 2-year overall survival outcomes in a multicentre, randomised, controlled, phase 2 trial. *Lancet Oncol.* 2016;17(11):1558-1568.
3. Larkin J, Minor D, D'Angelo S, et al. Overall Survival in Patients With Advanced Melanoma Who Received Nivolumab Versus Investigator's Choice Chemotherapy in CheckMate 037: A Randomized, Controlled, Open-Label Phase III Trial. *J Clin Oncol.* 2018;36(4):383-390.
4. Robert C, Schachter J, Long GV, et al. Pembrolizumab versus Ipilimumab in Advanced Melanoma. *N Engl J Med.* 2015;372(26):2521-2532.
5. Hodi FS, O'Day SJ, McDermott DF, et al. Improved survival with ipilimumab in patients with metastatic melanoma. *N Engl J Med.* 2010;363(8):711-723.
6. Robert C, Thomas L, Bondarenko I, et al. Ipilimumab plus dacarbazine for previously untreated metastatic melanoma. *N Engl J Med.* 2011;364(26):2517-2526.
7. Hodi FS, Lee S, McDermott DF, et al. Ipilimumab plus sargramostim vs ipilimumab alone for treatment of metastatic melanoma: a randomized clinical trial. *JAMA.* 2014;312(17):1744-1753.
8. Ribas A, Kefford R, Marshall MA, et al. Phase III randomized clinical trial comparing tremelimumab with standard-of-care chemotherapy in patients with advanced melanoma. *J Clin Oncol.* 2013;31(5):616-622.
9. Fehrenbacher L, Spira A, Ballinger M, et al. Atezolizumab versus docetaxel for patients with previously treated non-small-cell lung cancer (POPLAR): a multicentre, open-label, phase 2 randomised controlled trial. *Lancet.* 2016;387(10030):1837-1846.
10. Rittmeyer A, Barlesi F, Waterkamp D, et al. Atezolizumab versus docetaxel in patients with previously treated non-small-cell lung cancer (OAK): a phase 3, open-label, multicentre randomised controlled trial. *Lancet.* 2017;389(10066):255-265.
11. Borghaei H, Paz-Ares L, Horn L, et al. Nivolumab versus Docetaxel in Advanced Nonsquamous Non-Small-Cell Lung Cancer. *N Engl J Med.* 2015;373(17):1627-1639.
12. Brahmer J, Reckamp KL, Baas P, et al. Nivolumab versus Docetaxel in Advanced Squamous-Cell Non-Small-Cell Lung Cancer. *N Engl J Med.* 2015;373(2):123-135.
13. Carbone DP, Reck M, Paz-Ares L, et al. First-Line Nivolumab in Stage IV or Recurrent Non-Small-Cell Lung Cancer. *N Engl J Med.* 2017;376(25):2415-2426.
14. Herbst RS, Baas P, Kim DW, et al. Pembrolizumab versus docetaxel for previously treated, PD-L1-positive, advanced non-small-cell lung cancer (KEYNOTE-010): a randomised controlled trial. *Lancet.* 2016;387(10027):1540-1550.
15. Langer CJ, Gadgeel SM, Borghaei H, et al. Carboplatin and pemetrexed with or without pembrolizumab for advanced, non-squamous non-small-cell lung cancer: a

© 2019 Wang Z-X et al. *JAMA Network Open.*

randomised, phase 2 cohort of the open-label KEYNOTE-021 study. *Lancet Oncol.* 2016;17(11):1497-1508.

16. Reck M, Rodriguez-Abreu D, Robinson AG, et al. Pembrolizumab versus Chemotherapy for PD-L1-Positive Non-Small-Cell Lung Cancer. *N Engl J Med.* 2016;375(19):1823-1833.
17. Lynch TJ, Bondarenko I, Luft A, et al. Ipilimumab in combination with paclitaxel and carboplatin as first-line treatment in stage IIIB/IV non-small-cell lung cancer: results from a randomized, double-blind, multicenter phase II study. *J Clin Oncol.* 2012;30(17):2046-2054.
18. Kang YK, Boku N, Satoh T, et al. Nivolumab in patients with advanced gastric or gastro-oesophageal junction cancer refractory to, or intolerant of, at least two previous chemotherapy regimens (ONO-4538-12, ATTRACTION-2): a randomised, double-blind, placebo-controlled, phase 3 trial. *Lancet.* 2017;390(10111):2461-2471.
19. Ferris RL, Blumenschein G, Jr., Fayette J, et al. Nivolumab for Recurrent Squamous-Cell Carcinoma of the Head and Neck. *N Engl J Med.* 2016;375(19):1856-1867.
20. Kwon ED, Drake CG, Scher HI, et al. Ipilimumab versus placebo after radiotherapy in patients with metastatic castration-resistant prostate cancer that had progressed after docetaxel chemotherapy (CA184-043): a multicentre, randomised, double-blind, phase 3 trial. *Lancet Oncol.* 2014;15(7):700-712.
21. Beer TM, Kwon ED, Drake CG, et al. Randomized, Double-Blind, Phase III Trial of Ipilimumab Versus Placebo in Asymptomatic or Minimally Symptomatic Patients With Metastatic Chemotherapy-Naive Castration-Resistant Prostate Cancer. *J Clin Oncol.* 2017;35(1):40-47.
22. Motzer RJ, Escudier B, McDermott DF, et al. Nivolumab versus Everolimus in Advanced Renal-Cell Carcinoma. *N Engl J Med.* 2015;373(19):1803-1813.
23. Reck M, Bondarenko I, Luft A, et al. Ipilimumab in combination with paclitaxel and carboplatin as first-line therapy in extensive-disease-small-cell lung cancer: results from a randomized, double-blind, multicenter phase 2 trial. *Ann Oncol.* 2013;24(1):75-83.
24. Reck M, Luft A, Szczesna A, et al. Phase III Randomized Trial of Ipilimumab Plus Etoposide and Platinum Versus Placebo Plus Etoposide and Platinum in Extensive-Stage Small-Cell Lung Cancer. *J Clin Oncol.* 2016;34(31):3740-3748.
25. Powles T, Duran I, van der Heijden MS, et al. Atezolizumab versus chemotherapy in patients with platinum-treated locally advanced or metastatic urothelial carcinoma (IMvigor211): a multicentre, open-label, phase 3 randomised controlled trial. *Lancet.* 2018;391(10122):748-757.
26. Bellmunt J, de Wit R, Vaughn DJ, et al. Pembrolizumab as Second-Line Therapy for Advanced Urothelial Carcinoma. *N Engl J Med.* 2017;376(11):1015-1026.

© 2019 Wang Z-X et al. *JAMA Network Open.*
